# High-throughput Screening of Sequence Elements Associated with RNA Localization

**DOI:** 10.34133/csbj.0107

**Published:** 2026-05-21

**Authors:** Xinquan Zeng, Yusen Lin, Meiting Cai, Jingxia Lin, Yongjun Zhang, Mingze Yao, Jiajian Zhou

**Affiliations:** ^1^ Dermatology Hospital, Southern Medical University, Guangzhou 510180, China.; ^2^ Institutes of Biomedical Sciences, Shanxi Provincial Key Laboratory for Medical Molecular Cell Biology, Key Laboratory of Chemical Biology and Molecular Engineering of Ministry of Education, Shanxi University, Taiyuan 030006, China.; ^3^ Guangdong Provincial Key Laboratory of Single-cell and Extracellular Vesicles, Southern Medical University, Guangzhou 510180, China.

## Abstract

RNA molecules localize to specific subcellular compartments to perform their biological functions correctly. However, the mechanisms underlying RNA transport and subcellular localization remain poorly understood. In this study, we introduced SRLE-seq (Screening RNA Localization Elements by Sequencing) to identify and prioritize functional sequence elements associated with RNA localization. Our approach successfully recovered known elements and identified 2 novel elements linked to nuclear retention by screening randomized *MALAT1* (metastasis-associated lung adenocarcinoma transcript 1) fragments. Within our screening library of 6-mer sequences, SRLE-seq identified 110 6-mers exhibiting functions related to nuclear retention and 49 6-mers associated with nuclear export. Notably, we found that the nuclear retention score derived from SRLE-seq improves predictions of RNA subcellular localization, achieving 72% accuracy when incorporated into a deep learning model. Further experiments demonstrated that these localization-associated 6-mers exert their effects through the regulation of RNA-binding proteins. Our study presents a highly efficient method for prioritizing RNA localization elements via sequencing, establishing a valuable resource for further investigation of RNA-binding protein-mediated RNA subcellular localization.

## Introduction

RNA molecules are transported to specific subcellular localizations after transcription and accurately perform their functions there, including the nucleus, cytoplasm, mitochondria, and endoplasmic reticulum [1–4]. In eukaryotes, protein-coding genes (e.g., mRNAs) are exported from the nucleus to the cytoplasm along with transcription and translated to a polypeptide in the endoplasmic reticulum [5–7]. In contrast, most noncoding RNAs (ncRNAs), particularly long noncoding RNAs (lncRNAs), are retained in the nucleus for transcriptional regulation in a context-dependent manner [8–11]. Therefore, the subcellular localization of RNAs is crucial for exerting a gene’s functions. RNA fluorescence in situ hybridization and dead Cas13a-mediated imaging-based methods have been widely used to study RNA functions, but they are low-efficient [4,12,13]. The biochemical fractionation methods (CeFra-seq) and proximity ligation methods (APEX-seq, CAP-seq, Halo-seq, and BAP-seq) enable the establishment of an RNA subcellular localization atlas by leveraging high-throughput RNA sequencing (RNA-seq) [14–18]. Recent high-resolution spatial single-cell RNA-seq enables screening thousands of RNA molecules at the single-cell level, accelerating the establishment of an RNA localization atlas [19–21]. However, the mechanism of RNA transport and subcellular localization requires further investigation [22–24].

Recent studies have shown that RNA-binding proteins (RBPs) and sequence motifs together drive the transportation of RNA molecules among different subcellular compartments [25–28]. Initially, Ross *et al.* [29] found that zipcode-binding protein binds to the *β-actin* mRNA (ACACCC) and drives its subcellular localization. Subsequently, Zuckerman and Ulitsky [30] showed that inefficient splicing drives the nuclear retention of lncRNAs. Alu repeats and U1 small nuclear ribonucleoproteins are involved in the nuclear localization of long RNAs in human cells [11,31–33]. Then, the researchers developed a high-throughput method to screen the sequence elements associated with the subcellular localization of linear and circular RNA [34,35]. Shen *et al.* developed REL-seq and mutREL-seq to screen the sequence elements associated with the subcellular localization of an RNA [33,36]. However, these methods either rely on the synthesis of 100-bp fragments or on the random fragmentation of sequences from a limited number of RNAs, due to the limited transfection efficiency. Thus, a comprehensive method for screening short motifs associated with RNA subcellular localization warrants further investigation.

In this study, we developed SRLE-seq (Screening RNA Localization Elements by Sequencing), a method for screening for functional RNA localization elements using high-throughput sequencing. SRLE-seq successfully decodes the sequence elements associated with RNA subcellular localization in the *MALAT1* (metastasis-associated lung adenocarcinoma transcript 1) gene and all 6-nt subsequences (6-mers). We will apply the scored 6-mers to enhance the prediction ability of a deep learning model and prioritize RBPs associated with RNA subcellular localization. It will establish a valuable resource for investigating RBP-mediated RNA subcellular localization.

## Materials and Methods

### Cell culture and transfection

HEK293T cells (HEK293 cells transformed with large T antigen, ATCC CRL-3216) were maintained in complete Dulbecco’s modified Eagle’s medium (DMEM), which consists of 89% DMEM basal medium supplemented with 10% fetal bovine serum and 1% penicillin/streptomycin solution. The cells were kept at 37 °C in a 5% CO₂ atmosphere. Routine testing for mycoplasma contamination was performed using polymerase chain reaction (PCR). The PEI (polyethylenimine) (ServiceBio, PEI40K) transfection was conducted when the cells reached 70% to 80% confluency. After transfection, the medium was replaced with complete DMEM, and the cells were maintained for 8 h. The transfected cells were then harvested 24 h posttransfection.

### Subcellular fractionation of HEK293T cells

The subcellular fractionation protocol for HEK293T cells was adapted from a previous study [37]. The cells were harvested from the culture dish using trypsin and then incubated at 37 °C, 5% CO₂ for 1 min. Then, the cells were centrifuged at 800g for 5 min and washed twice with 1 ml of PBS. Typically, we obtained approximately 2 × 10^7^ cells from a 10-cm dish. Subsequently, the cells were then resuspended in 1 ml of lysis buffer (10 mM tris-Cl, pH 8.0, 140 mM NaCl, 1.5 mM MgCl₂, 0.5% Igepal, 2 mM vanadyl ribonucleoside diethyl phosphorocyanidate [DEPC] H_2_O) and incubated on ice for 4 min. Cell integrity was assessed by trypan blue staining to detect cytoplasmic release and nuclear membrane integrity. For the cytoplasmic fraction, the remaining suspension (~1 ml) was centrifuged at 800g, 4 °C for 3 min; 800 μl of the supernatant was transferred to a new tube, and then 3 rounds of centrifugation at 13,400g, 4 °C for 10 min were performed to yield 400 μl of purified cytoplasmic extract. For the nuclear fraction, the pellet was resuspended in 1 ml of Lysis SD Buffer (Lysis Buffer with 0.5% sodium deoxycholate), gently pipetted 5 to 7 times, and incubated on ice for 5 min; then, centrifugation was performed at 1,000g, 4 °C for 3 min to obtain the nuclear fraction by complete supernatant removal.

### Plasmid library construction

The N1-HBB plasmid was constructed from pEGFP-N1 (Clontech) by linearizing the backbone and Gibson assembly [38], as described in our previous study [39] (Fig. S1A and B). Briefly, the hemoglobin subunit beta (*HBB*) genomic sequence and *HBB* transcript were amplified using primers that contained homology arms complementary to the backbone, respectively. The amplified sequences were then integrated into the backbone via the Gibson assembly kit (RK21020). For the N1-HBB-MALAT1 library, genomic DNA of *MALAT1* was amplified and fragmented using deoxyribonuclease I for 20 min; the fragments were then end-repaired, dA-tailed, and TA-ligated with adaptor F/R oligonucleotides. Finally, the adaptor-ligated fragments were PCR-amplified, purified, and recombined into the 3’ untranslated region (3’ UTR) of N1-HBB via Gibson assembly (Fig. S2A to C). For the N1-HBB-6-mer library, 6-nt random sequences flanked by 5’ and 3’ homology arms of the *HBB* 3’ UTR were synthesized and subsequently cloned into the N1-HBB backbone at the *HBB* 3’ UTR (Fig. S3). All oligo information is listed in Table S1.

### SRLE-seq

The nuclear and cytoplasmic fractions were subjected to RNA extraction using TRIzol reagent (T9424, Sigma). The extracted RNA was quantified with a Qubit RNA assay (Q32851, Thermo Fisher), and 1 μg of RNA was reverse-transcribed into complementary DNA (cDNA) using EasyScript One-Step RT-PCR SuperMix (AE411-02, Transgene, Beijing, China) according to the manufacturer’s instructions. Subsequently, the 6-mer and the flanking sequences were amplified by PCR for 25 cycles, using 5 μl of cDNA as the template. The PCR products were then purified and quantified, and 1 μg of each purified product was submitted for high-throughput sequencing on the MGI DNBSEQ-T7 platform. Additionally, the perturbation in *HBB* subcellular distribution upon insertion of fragments or 6-mers was quantified by quantitative PCR (qPCR), using 5 μl of the remaining cDNA as the template. For each library prepared for sequencing, samples were collected in triplicate biological replicates to ensure the reliability of our experiments. To quantify *HBB* abundance across subcellular localizations, we performed qPCR on cDNA derived from nuclear and cytoplasmic fractions, using N1-HBB, which carries a specific insertion. The sequence information for PCR primers is provided in Table S2.

### SRLE-seq data analysis

The low-quality and adapter sequences were trimmed using Trimmomatic with default parameters [40]. We defined a read as valid if it contains the constant 10-bp flanking region of an inserted sequence. For the 6-mer library, raw counts and counts per million reads (normalized by each sample’s sequencing depth) of fragment sequences (6-mers) were calculated in the nuclear and cytoplasmic fractions to evaluate subcellular distribution. The log2 ratio of the counts per million reads of an inserted sequence element in the nucleus to that in the cytoplasm is defined as NRS (nuclear retention score), similar to a previous study [41]. It represents the likelihood of its effect on RNA subcellular localization. The raw counts of 6-mers in 3 biological replicates of the nuclear and cytoplasmic fractions were analyzed with DESeq2 [42] for sequencing-depth normalization, dispersion estimation, and differential expression analysis of 6-mers using the Wald test with multiple-testing correction. Subsequently, the nuclear- and cytoplasm-enriched sequence elements were identified with NRS >= 0.58, *P* value < 0.05 and NRS <= −0.58, *P* value < 0.05, respectively. Regarding the N1-HBB-MALAT1 library, we used a similar scheme to identify the nuclear- and cytoplasm-enriched sequence elements as the 6-mer library. In addition, the nuclear- and cytoplasm-enriched sequence elements were mapped back to the reference genome using bowtie2 [43], and we manually retrieved the specific regions associated with nuclear retention of the *MALAT1* gene by comparing the coverage track using Integrative Genome Viewer (IGV) [44].

### Identification of nuclear- and cytoplasm-enriched RNAs across 12 cell types

The PolyA^+^ RNA-seq dataset, obtained from the nuclear or cytoplasmic fractions of 12 cell types, was downloaded from the ENCODE project [45] (Table S3). It was generated using the same protocol to mitigate heterogeneity in next-generation sequencing data. The RNA-seq data were processed as described in previous studies [37,39]. In brief, adapter sequences and low-quality reads were trimmed from 3′ to 5′ ends. Reads shorter than 36 bp were discarded, and the remaining reads were mapped to the human genome (hg38) using Hisat2 [46]. The raw count for each gene locus was estimated using featureCounts. Gene expression abundance was reported in fragments per kilobase million (FPKM). The log_2_(fold change) was calculated by comparing the FPKM values from the nuclear and cytoplasmic fractions. RNAs were classified as nuclear-enriched if the expression level of the nuclear fraction exceeded a log2 fold change (log_2_[fold change]) threshold compared with the cytoplasmic fraction (log_2_[fold change] >= 1) across 12 cell types. Similarly, we identified cytoplasmic-enriched RNAs using log_2_(fold change) <= −1 as a cutoff.

### Establishment of an NRS-guided deep learning model for RNA subcellular localization prediction

To train the NRS-guided deep learning model, 80% of the nuclear-enriched and cytoplasmic-enriched RNAs were subjected to the training step, while the remaining 20% were subjected to the testing step. The NRS values of 4,096 6-mers or the average NRS of a 6-nt consecutive region (NRS avg.) were assigned to RNA transcripts using a 6-nt sliding window and 1-nt per step. Each transcript was then transformed into an *n* × *m* feature matrix via an encoding process (*n* is the length of the longest transcript; *m* is a hyperparameter set to 256). This matrix was fed into a multilayer Transformer model [47] to capture dependencies among 6-mers positioned at different locations within a transcript. Notably, we added a special classification (CLS) token to the feature matrix, which serves as a vector to aggregate NRS-guided sequence information for classification. Ultimately, the CLS token is used to classify transcripts as nuclear- or cytoplasmic-enriched. We applied the epoch-accuracy curve to assess the predictive power of the NRS values and the epoch-loss curve to address overfitting. In the training process, we found that the loss value of the test began to increase after epoch 6, indicating overfitting, so we stopped training to avoid it (Fig. S4). For the comparison analysis, we generated a randomized NRS of 6-mers with the same distribution (Rand. and Rand. avg.), and the baseline model included only 6-mer information (Baseline). This randomized NRS was evaluated using a methodology similar to that described above.

### Identification of subcellular localization-associated RBPs

The NRS values were assigned to RNA transcripts as described above. Subsequently, we identified subcellular enrichment-associated peaks using the following cutoffs: NRS >= 0.5 for nuclear peaks and NRS <= −0.5 for cytoplasmic peaks. These peaks were then subjected to RBP binding motif enrichment analysis using DREME [48], and the enriched motifs were mapped to their corresponding RBPs using TOMTOM [48]. The RBPs that significantly matched the motifs were identified as potential regulators bound to the motifs enriched in either nuclear or cytoplasmic peaks. Short interfering RNA or short hairpin RNA knockdowns were performed for the 3 selected RBPs, followed by cell fractionation and RNA-seq with 3 replicates. The RNA-seq dataset derived from the nuclear or cytoplasmic fraction was processed as described in our previous study [39]. First, the log_2_FC of each gene’s expression in the nuclear fraction relative to the cytoplasmic fraction was calculated for both knockdown and control samples. Then, the change in subcellular localization was evaluated using the log_2_FC difference between knockdown and control samples (Δlog_2_FC) and Student *t* test. The “Shift to nuclear in KD” effect is defined as Δlog_2_FC > 0.58 and *P* value < 0.05, while the “Shift to cytoplasm in KD” is defined as Δlog_2_FC < −0.58 and *P* value < 0.05. Finally, we used a volcano plot with a density heatmap to visualize changes in localization for each knockdown experiment with the Python Matplotlib package.

### Statistical analysis

Data were analyzed using GraphPad Prism (version 8; GraphPad Software, San Diego, CA). The data were represented as mean ± SD. All tests were 2-sided, and *P* < 0.05 was considered statistically significant.

## Results

### SRLE-seq: Screening RNA localization elements by high-throughput sequencing

**Fig. 1.**
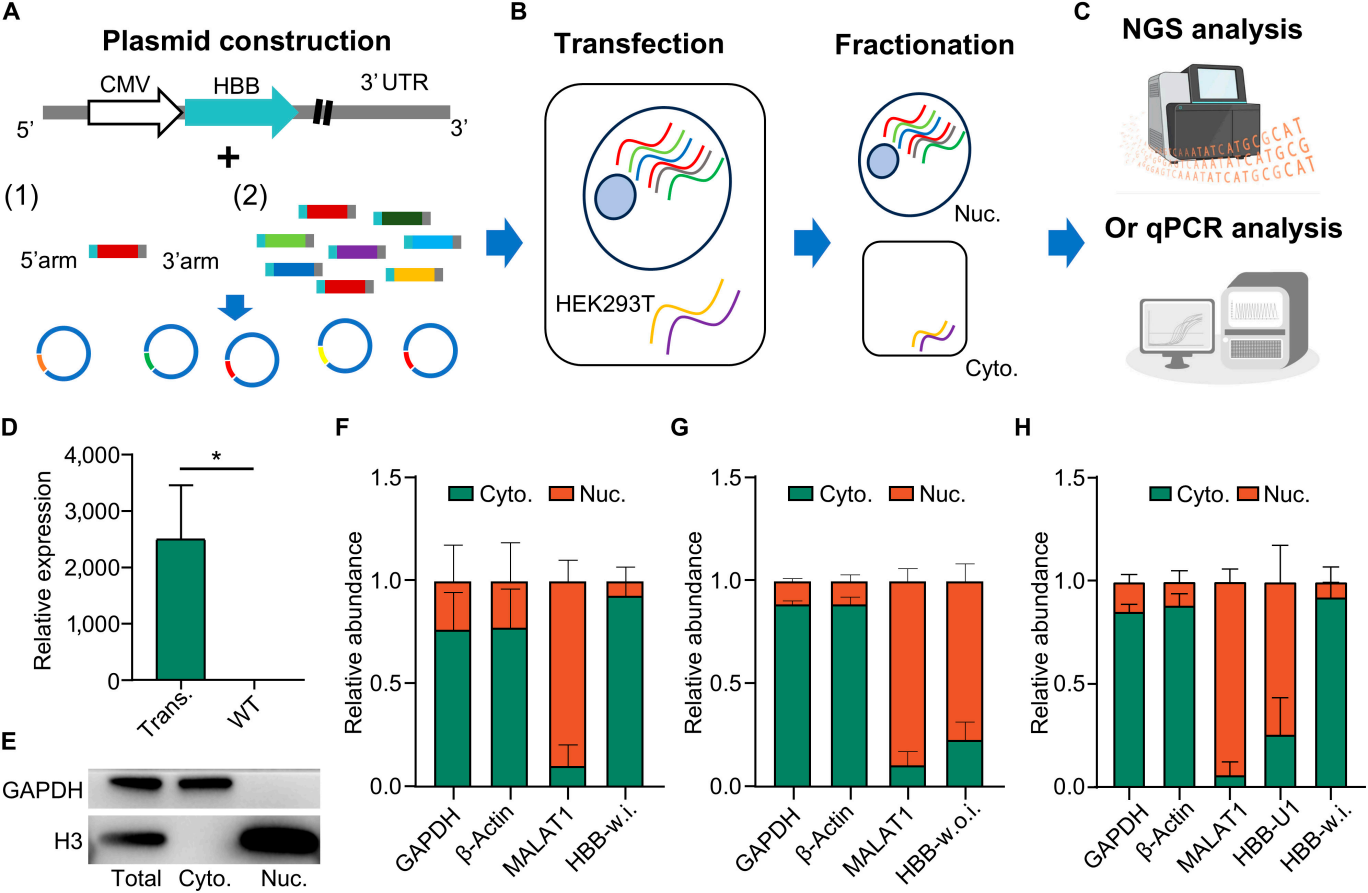
SRLE-seq: Screening RNA localization elements by high-throughput sequencing. (A) N1-HBB plasmid construction. The sequence elements are inserted into the 3’ untranslated region (3’ UTR) of the hemoglobin subunit beta (*HBB*) gene via Gibson assembly. (B) N1-HBB plasmid library transfection in HEK293T (HEK293 cells transformed with large T antigen), subcellular fractionation, followed by RNA extraction and reverse transcription. (C) Profiling the subcellular distribution of *HBB* transcript through the log2 ratio of the sequence element count using the high-throughput sequencing or quantitative polymerase chain reaction (qPCR) analysis in the nucleus and cytoplasm fraction. (D) The exogenous *HBB* is constitutively expressed after N1-HBB transfection. (E) Western blot showed that the nuclear and cytoplasmic fractions are separated. While glyceraldehyde phosphate dehydrogenase (*GAPDH*) is predominantly in the cytoplasm, H3 is exclusively in the nuclear fraction. (F and G) *HBB* genes with introns (HBB-w.i.) showed predominant cytoplasmic localization compared with *GAPDH*, *β-actin*, and *MALAT1* (metastasis-associated lung adenocarcinoma transcript 1); conversely, the *HBB* gene without introns (HBB-w.o.i.) showed nuclear localization. (H) *HBB* transcript shifted from the cytoplasm to the nucleus upon the U1 motif insertion in the 3’ UTR. *P < 0.05. CMV, cytomegalovirus; Trans., with N1-HBB transfected; Total, the whole cell; Cyto., cytoplasm; Nuc., nucleus; NGS, next-generation sequencing.

RNA molecules precisely exert their functions in a spatially precise manner across various subcellular localizations, yet the sequence elements associated with RNA subcellular localization require further investigations [11,22,49]. To address this gap, we introduced SRLE-seq, a high-throughput method that prioritizes subcellular localization-associated sequence elements in RNA. SRLE-seq consists of 3 main parts: (a) Plasmid construction. We constructed a plasmid containing a cytomegalovirus promoter, the *HBB* gene, and a 3’ UTR element for the overexpression of the target sequence element, referred to as N1-HBB. A plasmid library is created by ligating various sequence elements into the 3’ UTR of N1-HBB using Gibson assembly (Fig. 1A and Fig. S1A). (b) Transfection and cell fractionation. The constructed library is transfected into target cell cultures for 24 h, followed by cell fractionation and RNA extraction (Fig. 1B). (c) RNA-abundant analysis. The RNA extracted from both nuclear and cytoplasmic fractions is reverse-transcribed. The sequence elements are then amplified and analyzed through high-throughput sequencing or quantified using qPCR. The log2 ratio of a specific element in the nucleus to that in the cytoplasm (NRS) indicates its potential role in influencing RNA subcellular localization (Fig. 1C). To validate the method, we applied SRLE-seq in the HEK293T cell line, which has high transfection efficiency and lacks *HBB* expression (Fig. 1D). Western blot analysis showed that glyceraldehyde 3-phosphate dehydrogenase (*GAPDH*) was exclusively present in the cytoplasm, while H3 was exclusively present in the nuclear fraction, confirming the effective separation of the cytoplasmic and nuclear components (Fig. 1E). Furthermore, we observed that the *HBB* fragment containing introns was predominantly located in the cytoplasm, as were *GAPDH* and *β-actin*. In contrast, the *HBB* without introns was predominantly nuclear and comparable to *MALAT1* (Fig. 1F and G). Therefore, we proceeded with the plasmid containing *HBB* with introns for subsequent experiments. To demonstrate the viability of SRLE-seq, we investigated the subcellular distribution of the *HBB* transcript with and without an insertion of a U1 motif (Fig. S2B), which has been reported to play a role in RNA nuclear retention [50]. Our results showed that *HBB*, *GAPDH*, and *β-actin* were predominantly localized in the cytoplasm, while *MALAT1* was found primarily in the nuclear fraction (Fig. 1H). In summary, our analysis demonstrated that SRLE-seq is an effective method for testing sequence elements that influence RNA subcellular localization.

### Identification of nuclear localization-associated elements in *MALAT1*

**Fig. 2.**
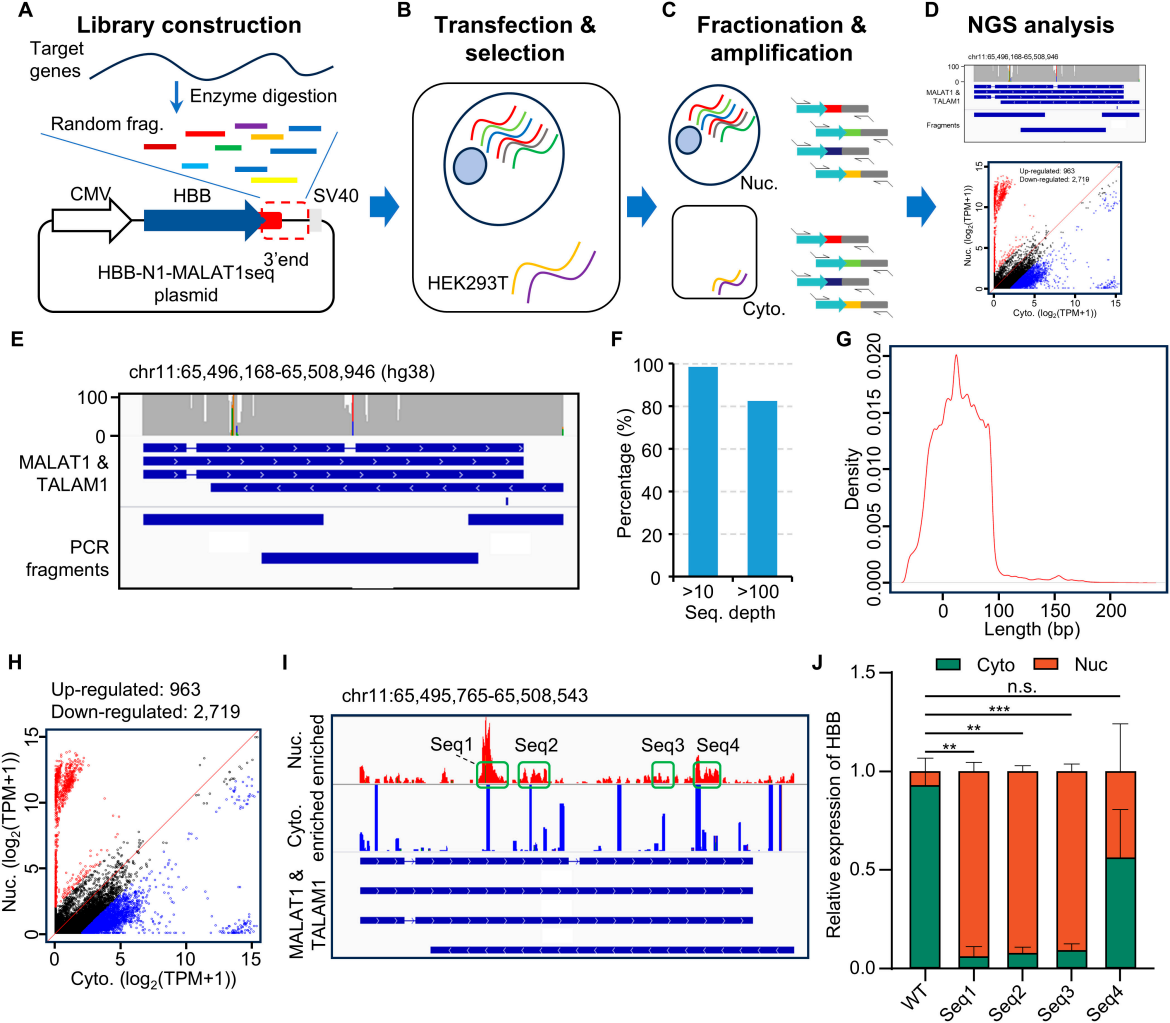
Identification of nuclear localization-associated elements in *MALAT1* (metastasis-associated lung adenocarcinoma transcript 1). (A) N1-HBB-MALAT1 library construction. *MALAT1* is reverse-transcribed and subjected to random fragmentation via enzymatic digestion. The 75- to 150-bp fragments were amplified and cloned into N1-HBB. (B) N1-HBB-MALAT1 library was transfected into HEK293T (HEK293 cells transformed with large T antigen). (C) Subcellular fractionation and target amplification of HEK293T cells. RNA is extracted separately from the cytoplasm and nucleus. (D) Profiling the subcellular distribution of different sequence elements using nuclear retention score (NRS) and manually retrieved the specific regions associated with nuclear retention of the *MALAT1* gene by comparing the coverage track using Integrative Genomics Viewer (IGV). (E) The coverage analysis of sequence elements on the *MALAT1* gene. (F) The bar chart shows that the *MALAT1* transcript is covered at least 10-fold. (G) The fragment length distribution of sequence elements in the *MALAT1* library. (H) The scatter plot shows the nuclear and cytoplasmic-enriched sequence elements in *MALAT1*. (I) Distribution of sequence elements in the *MALAT1* transcript of the nuclear and cytoplasmic fractions. (J) The subcellular distribution of hemoglobin subunit beta (*HBB*) transcript in N1-HBB-WT, N1-HBB-Seq1, N1-HBB-Seq2, N1-HBB-Seq3, and N1-HBB-Seq4. **P < 0.01, ***P < 0.001. n.s., not significant.

*MALAT1* plays an important role in nuclear transcriptional regulation, and several sequence elements have been identified that confer strong nuclear retention [51,52]. Therefore, our goal was to test whether SRLE-seq could recover known elements and discover new elements associated with nuclear retention. We amplified the *MALAT1* genomic sequence, randomly fragmented it, and cloned the sequence elements into N1-HBB, as illustrated in Fig. 2A (see Fig. S1B and C for details). We then applied SRLE-seq to identify elements associated with the nuclear localization of *MALAT1* (Fig. 2A to D). Unsurprisingly, the detected fragments covered nearly all *MALAT1* loci, with 95% of *MALAT1* showing a sequencing depth greater than 10 (Fig. 2E and F). The lengths of the detected fragments ranged from 30 to 100 bp (Fig. 2G), which is sufficient for an RBP binding event [53,54]. Furthermore, we identified 963 nuclear retention elements and 2,719 cytoplasm localization elements by comparing the observed abundances in nuclear and cytoplasmic fractions (Fig. 2H). The enrichment ratio analysis revealed 4 sequence elements with higher NRSs (Fig. 2I). Two of these elements were validated in previous studies [52,55], while seq2 and seq4 were identified for the first time in our study. Notably, we observed that the *HBB* transcript shifted from the cytoplasm to the nuclear fraction upon the insertion of seq1, seq2, and seq3 into the N1-HBB plasmid (Fig. 2J and Table S4). These results demonstrate that SRLE-seq effectively identifies subcellular localization-associated elements in a high-throughput manner.

### Screening subcellular localization-associated 6-mers

**Fig. 3.**
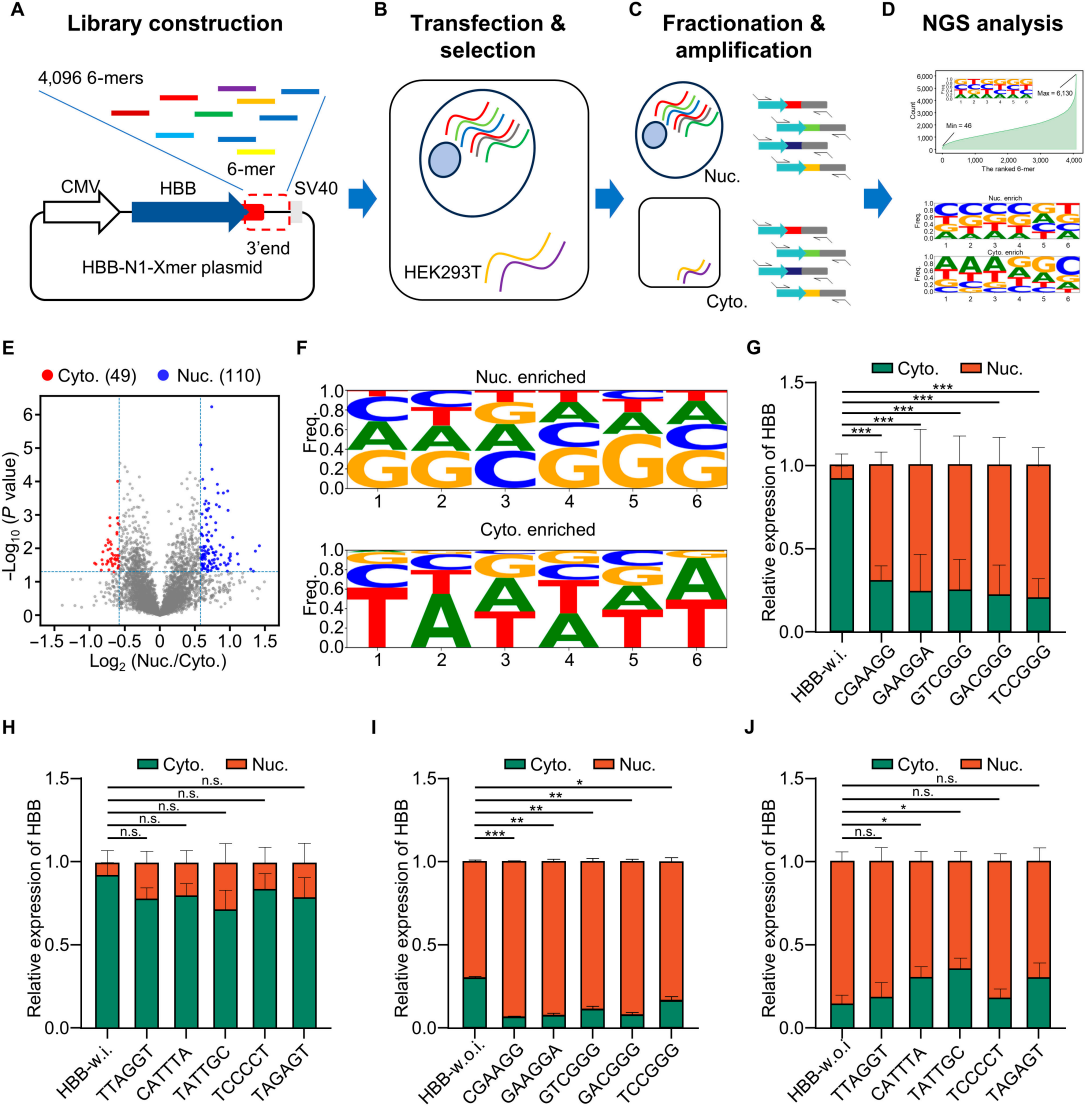
Screening subcellular localization-associated 6-mers. (A) N1-HBB-6-mer library construction. The linearization primers, which included recombination arms and randomized 6-mers, were applied to linearize the N1-HBB; it introduces a 6-mer element in the 3’ untranslated region (3’ UTR) of the hemoglobin subunit beta (*HBB*) gene. (B) Plasmid transfection of HEK293T cells (HEK293 cells transformed with large T antigen). After 24 h of transfection, *HBB* RNA is expressed and undergoes intracellular localization. (C) Subcellular fractionation and target amplification of HEK293T cells. RNA is extracted separately from the cytoplasm and nucleus. (D) Profiling the subcellular distribution of different sequence elements using nuclear retention score (NRS). (E) The volcano plot showed the nuclear- (110) and cytoplasmic-enriched (49) 6-mers. (F) Motif enrichment analysis of 6-mers associated with RNA subcellular localization. (G and H) Quantitative polymerase chain reaction (qPCR) experiment validated the subcellular localization of *HBB* transcripts with 10 6-mers insertions in the 3’ UTR of *HBB*-w.i., respectively. Nuclear-enriched: Nuc1 (CGAAGG), Nuc2 (GAAGGA), Nuc3 (GTCGGG), Nuc4 (GACGGG), and Nuc5 (TCCGGG); cytoplasmic-enriched: Cyto1 (TTAGGT), Cyto2 (CATTTA), Cyto3 (TATTGC), Cyto4 (TCCCCT), and Cyto5 (TAGAGT). *P < 0.05, **P < 0.01, ***P < 0.001. n.s., not significant. (I and J) qPCR experiment validated the subcellular localization of *HBB* transcripts with 10 6-mers insertions in the 3’ UTR of *HBB*-w.o.i., respectively. Nuclear-enriched: Nuc1 (CGAAGG), Nuc2 (GAAGGA), Nuc3 (GTCGGG), Nuc4 (GACGGG), and Nuc5 (TCCGGG); cytoplasmic-enriched: Cyto1 (TTAGGT), Cyto2 (CATTTA), Cyto3 (TATTGC), Cyto4 (TCCCCT), and Cyto5 (TAGAGT). *P < 0.05, **P < 0.01, ***P < 0.001. n.s., not significant.

Next, we expanded our testing to include all 6-mers using SRLE-seq, as RBPs bind to specific motifs that are typically 4 to 8 bp long, facilitating the nuclear export of RNAs [33,56–59]. To this end, we designed a degenerate primer containing all possible 6-mers to linearize the plasmid and generate a synthetic library (6-mer-lib) via Gibson assembly (Fig. 3A and Fig. S3A). The 6-mer-lib was then transiently transfected into HEK293T cells. After transfection, RNA was extracted from both the nucleus and cytoplasm, followed by amplification of the 6-mers and high-throughput sequencing (Fig. 3B and C). The NRS of a 6-mer indicates its potential role in promoting RNA subcellular localization in nuclear retention and export (Fig. 3D). In the 6-mer library, we detected all 6-mers with more than 10-fold coverage, achieving a median coverage of 2,541-fold (Fig. S3B and C). From our analysis, we identified 110 6-mers with nuclear retention functions and 49 6-mers associated with RNA nuclear localization (Fig. 3E and Table S5). Motif analysis indicated a significant enrichment of GC bases in the nuclear-enriched 6-mers, while the cytoplasmic-enriched 6-mers displayed a pronounced enrichment of AT bases (Fig. 3F). Interestingly, we found that some 6-mers have been validated to promote RNA nuclear retention or export (Table S6). We then selected the top 5 nuclear-localizing 6-mers ranked by NRS for validation: Nuc1 (CGAAGG), Nuc2 (GAAGGA), Nuc3 (GTCGGG), Nuc4 (GACGGG), and Nuc5 (TCCGGG). Insertion of these 6-mers into the 3’ UTR of *HBB* shifted the distribution of the *HBB* transcript from the cytoplasm to the nucleus in both *HBB* constructs with and without introns (Fig. 3G and I). Notably, the top 5 cytoplasmic-localizing 6-mers did not affect the cytoplasmic localization of the *HBB* construct with introns (Fig. 3H), but these 6-mers affected the cytoplasmic localization of the *HBB* construct without introns (Fig. 3J). We speculated that alternative splicing plays an important role in promoting nuclear export and mitigating the effects of the cytoplasmic-localizing 6-mers on the *HBB* construct with introns. Overall, our analyses demonstrated that SRLE-seq effectively identifies 6-mers associated with subcellular localization.

### SRLE-seq enhances RNA subcellular localization prediction via an NRS-guided deep learning model

**Fig. 4.**
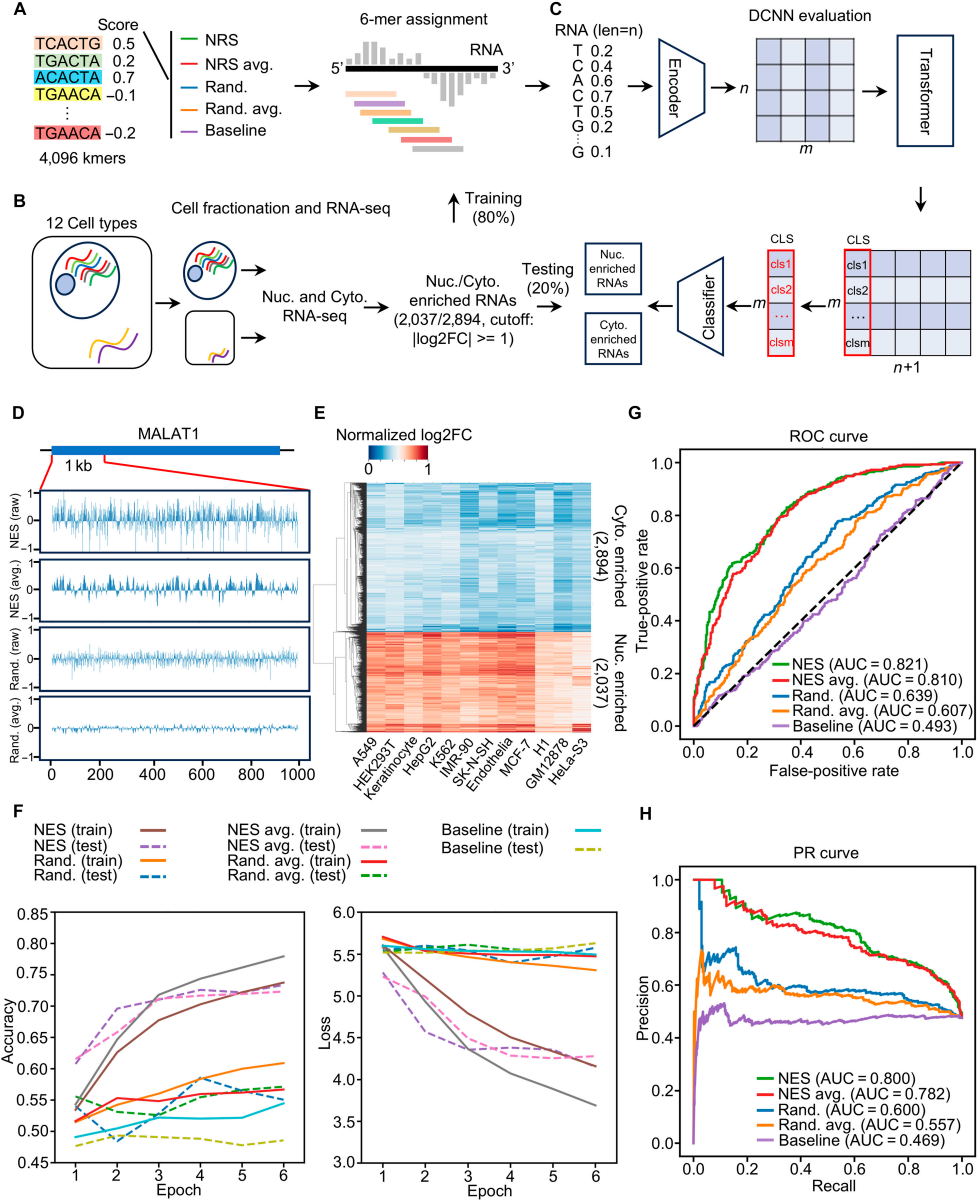
Screening RNA Localization Elements by Sequencing (SRLE-seq) enhances RNA subcellular localization prediction via a nuclear retention score (NRS)-guided deep learning model. (A) The workflow for assigning 6-mers across the entire transcript using SRLE-seq-derived NRS or randomized NRS. (B) The pipeline for identifying RNA enriched in the nucleus and cytoplasm across 12 cell types. (C) The design of the NRS-guided deep learning model used for classifying nuclear and cytoplasmic RNAs. (D) A snapshot showing the NRS signal (including raw NRS, average NRS, randomized raw NRS, and randomized average NRS) in a fragment of the *MALAT1* (metastasis-associated lung adenocarcinoma transcript 1) transcript. (E) The heatmap displays the expression levels of nuclear- and cytoplasmic-enriched RNAs. (F) The epoch-accuracy curve showed that the raw NRS and the average NRS were more informative for predicting RNA subcellular localization than the randomized NRS. (G and H) The receiver operating characteristic (ROC) and precision-recall (PR) curve show that the predictive power of NRS derived from SRLE-seq is better than the random and baseline models.

Next, we aim to investigate whether the NRS derived from SRLE-seq can improve RNA subcellular localization predictions using a deep learning model. To do this, we assigned both NRS and randomized NRS values of 4,096 6-mers to RNA transcripts using a 6-nt sliding window and 1 nt per step (Fig. 4A). When we compared with the randomized NRS, we observed many enriched peaks with NRS > 0 in the *MALAT1* transcript using both raw and average NRS, suggesting that the NRS derived from SRLE-seq reflects the importance of specific regions associated with RNA subcellular localization or RBP-binding events (Fig. 4D). Additionally, we examined transcriptomic datasets from 12 different cell lines using cell-fractionation RNA-seq from the ENCODE project [45]. Our analyses only retained 2,037 nuclear-enriched RNAs and 2,894 cytoplasmic-enriched RNAs across 12 cell lines to eliminate the cell type-specific effect on RNA subcellular localization (Fig. 4B and Table S7). These data were used to train (80%) and test (20%) a model to predict RNA subcellular localization. Specifically, all transcripts were encoded using NRS or randomized NRS; classification (CLS) tokens were calculated using a Transformer model; and transcripts were classified as nuclear-enriched or cytoplasmic-enriched RNAs (Fig. 4C). We used the epoch-accuracy curve to assess the predictive power of the NRS values and the epoch-loss curve to monitor for overfitting (Fig. S4). Notably, the raw NRS and average NRS achieved an accuracy of approximately 0.72 compared with the randomized NRS and the baseline model, which included only k-mer information, indicating that SRLE-seq enhances RNA subcellular localization predictions via an NRS-guided deep learning model (Fig. 4F). Additionally, the receiver operating characteristic) and precision-recall curve analyses showed similar results, supporting the predictive power of NRS derived from SRLE-seq (Fig. 4G and H). In summary, our analyses confirm that SRLE-seq effectively quantifies the functional contributions of 6-mers to RNA subcellular localization.

### Subcellular localization-associated 6-mers exert their functions through RBP-mediated regulation

**Fig. 5.**
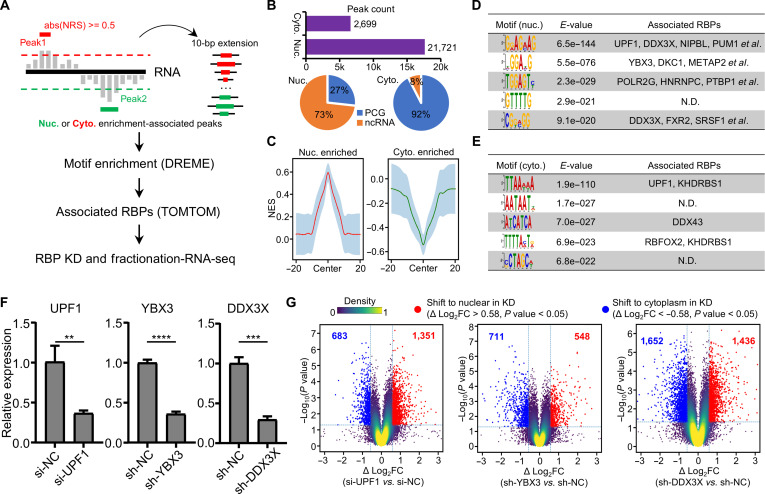
Subcellular localization-associated 6-mers exert their functions through RNA-binding protein (RBP)-mediated regulation. (A) The workflow used to identify RBPs that interact with RNA motifs linked to subcellular distribution. (B) The bar plot illustrates the number of peaks associated with the nuclear and cytoplasmic compartments, while the pie chart depicts the percentage of peaks derived from protein-coding genes (PCGs) and noncoding RNAs (ncRNAs). (C) The metagene plot displays the nuclear retention score (NRS) signal in the proximal region of peaks enriched in either the nucleus or the cytoplasm. (D and E) The 5 most enriched motifs along with their corresponding RBPs in the nuclear-enriched peaks (D) or cytoplasmic-enriched peaks (E). (F) Reverse transcription-quantitative polymerase chain reaction (RT-qPCR) results demonstrated the effects of short interfering RNA (siRNA) or short hairpin RNA (shRNA) knockdown of UPF1, YBX3, and DDX3X. (G) The volcano plots with a density heatmap present the changes of RNA subcellular distribution following the knockdown of UPF1, YBX3, and DDX3X. *P < 0.05, **P < 0.01, ***P < 0.001. N.D., not detected.

Previous studies have established RBPs as key mediators of RNA subcellular partitioning [22,27,28]. Therefore, we sought to elucidate potential interactions between RNA and RBPs to further explore the functions of 6-mers. We identified subcellular enrichment-associated peaks using the following cutoffs: NRS >= 0.5 for nuclear peaks and NRS <= −0.5 for cytoplasmic peaks. These peaks were then subjected to RBP binding motif enrichment analysis using DREME, and the enriched motifs were mapped to their corresponding RBPs using TOMTOM (Fig. 5A). As a result, we identified 2,699 peaks associated with cytoplasmic enrichment and 21,721 peaks associated with nuclear enrichment (Fig. 5B, top panel and Fig. 5C). A significant portion of the cytoplasmic-enrichment peaks originated from protein-coding genes, while a majority of the nuclear-enrichment peaks were derived from ncRNAs, indicating the reliability of our analysis pipeline (Fig. 5B, bottom panel). Further motif enrichment analysis revealed that the top 5 nuclear-enriched motifs were potentially bound by UPF1, DDX3X, NIPBL, PUM1, YBX3, DKC1, and HNRNPC (Fig. 5D) and the top 5 cytoplasmic-enriched motifs were potentially bound by UPF1, KHDRBS1, DDX43, and RBFOX2 (Fig. 5E). We subsequently selected UPF1, YBX3, and DDX3X for validation of their roles in RNA subcellular localization through knockdown experiments and cell-fractionation RNA-seq (Fig. 5F and G and Table S8). Notably, we observed that RNAs accumulated in the nucleus upon UPF1 knockdown, suggesting that UPF1 facilitates nuclear RNA export. Interestingly, UPF1 knockdown significantly increases the expression of Nonsense-mediated mRNA decay-target genes in whole-cell RNA-seq but does not affect the subcellular distribution of canonical Nonsense-mediated mRNA decay targets (Fig. S5). Additionally, RNAs are distributed in the cytoplasm upon YBX3 or DDX3X knockdown, suggesting that YBX3 or DDX3X helps retain RNAs in the nucleus, consistent with the motif enrichment analysis results. Overall, our analysis indicates that 6-mers associated with subcellular localization may exert their effects through RBP-mediated regulation.

## Discussions

SRLE-seq is a method designed for screening functional RNA localization elements using high-throughput sequencing. It focuses on identifying sequence elements associated with RNA subcellular localization in *MALAT1* and 6-mers. The scored 6-mers improved the prediction of RNA subcellular localization when applied to an NRS-guided deep learning model. Additionally, our analysis provided an alternative method for studying RNA subcellular localization mediated by RBPs.

RBPs and sequence motifs collaborate to orchestrate RNA trafficking within a cell [25–28]. Traditionally, researchers have used truncated sequences to identify functional motifs associated with RNA subcellular localization. However, this loss-of-function approach often suffers from low throughput and may disrupt the overall RNA structural context [60–62]. While synthetic libraries combined with high-throughput sequencing enable “gain-of-function” screens of thousands of candidate sequences simultaneously [32,33,63], they cannot encompass the entire transcriptome. We hypothesize that the interaction between sequence elements and RBPs is primarily mediated by short motifs (4 to 8 nt), with adjacent sequences also contributing to these interactions. Therefore, SRLE-seq screens focus on 6-mers associated with RNA subcellular localization, cover all sequence motifs in the transcriptome, and systematically conduct gain-of-function screens incorporating molecular engineering and high-throughput sequencing.

Previous studies used long synthetic fragments or stochastic cDNA fragmentation (ranging from 100 to 200 nt) for screening. However, these methods limit the resolution needed to pinpoint discrete regulatory elements within full-length transcripts and hinder the discovery of sequence motif–RBP interactions [32,33,35]. To address these limitations, we developed SRLE-seq, which screens functional RNA localization elements using a comprehensive library of synthetic 6-mers. This approach allows us to separate sequence-specific effects from the broader transcriptomic context, resulting in a detailed map of nuclear and cytoplasmic localization signals. Numerous studies have demonstrated that the transcript sequence features provide valuable information for predicting RNA subcellular localization [64–66]. Our research revealed that a deep learning model, guided by NRS and incorporating 6-mer NRS alongside transcript-based sequence features, achieved a predictive accuracy exceeding 0.72 for distinguishing nuclear-enriched from cytoplasmic-enriched RNAs across 12 human cell lines. Furthermore, we identified the peaks associated with subcellular enrichment using NSE, and the enriched motifs from these peaks were successfully linked to specific RBPs. Collectively, our study presents an alternative high-throughput method for screening functional RNA localization elements and their associated RBPs.

In the current study, several limitations can be addressed further: (a) The 6-mer library focuses solely on short motifs related to subcellular localization, neglecting the analysis of longer regulatory sequences and the combination of multiple 6-mers. (b) SRLE-seq investigates only the effects of sequence elements on the subcellular localization of synthetic *HBB* genes and requires further loss-of-function validation at a genome-wide level. Conducting an integrative analysis that combines a loss-of-function study using the CRISPR-KO screening system with SRLE-seq will help clarify the molecular mechanisms by which sequence elements regulate RNA subcellular localization.

## Data Availability

The source code is available on GitHub (https://github.com/lysovosyl/SRLE-seq), and the data are available in the Genome Sequence Archive (GSA) (HRA016642, https://ngdc.cncb.ac.cn/gsa-human/browse/HRA016642).
