# Supplementary material for: High-throughput Screening of Sequence Elements Associated with RNA Localization
Source: Comput Struct Biotechnol J. 2026 May 21;35(1):0107. doi: 10.34133/csbj.0107 (PMC13191086; doi:10.34133/csbj.0107)
Supplement: Supplementary 1 — Figs. S1 to S5 Tables S1 to S8 [file csbj.0107.f1.zip › Suppl.figures.v4.2.pdf]

SUPORTING INFORMATION:

High-throughput screening of sequence elements associated with RNA localization

Xinquan Zeng[1†], Yusen Lin[1†], Meiting Cai[1], Jingxia Lin[1], Yongjun Zhang[1], Mingze Yao[2], Jiajian Zhou[1,3*]

1. Dermatology Hospital, Southern Medical University, Guangzhou, 510180, China.

2. Institutes of Biomedical Sciences, Shanxi Provincial Key Laboratory for Medical Molecular Cell Biology, Key Laboratory of Chemical Biology and Molecular Engineering of Ministry of Education, Shanxi University, Taiyuan 030006, China.

3. Guangdong Provincial Key Laboratory of Single-cell and Extracellular Vesicles, Southern Medical University, Guangzhou, 510180, China.

Supplemental Figures

Figure S1.

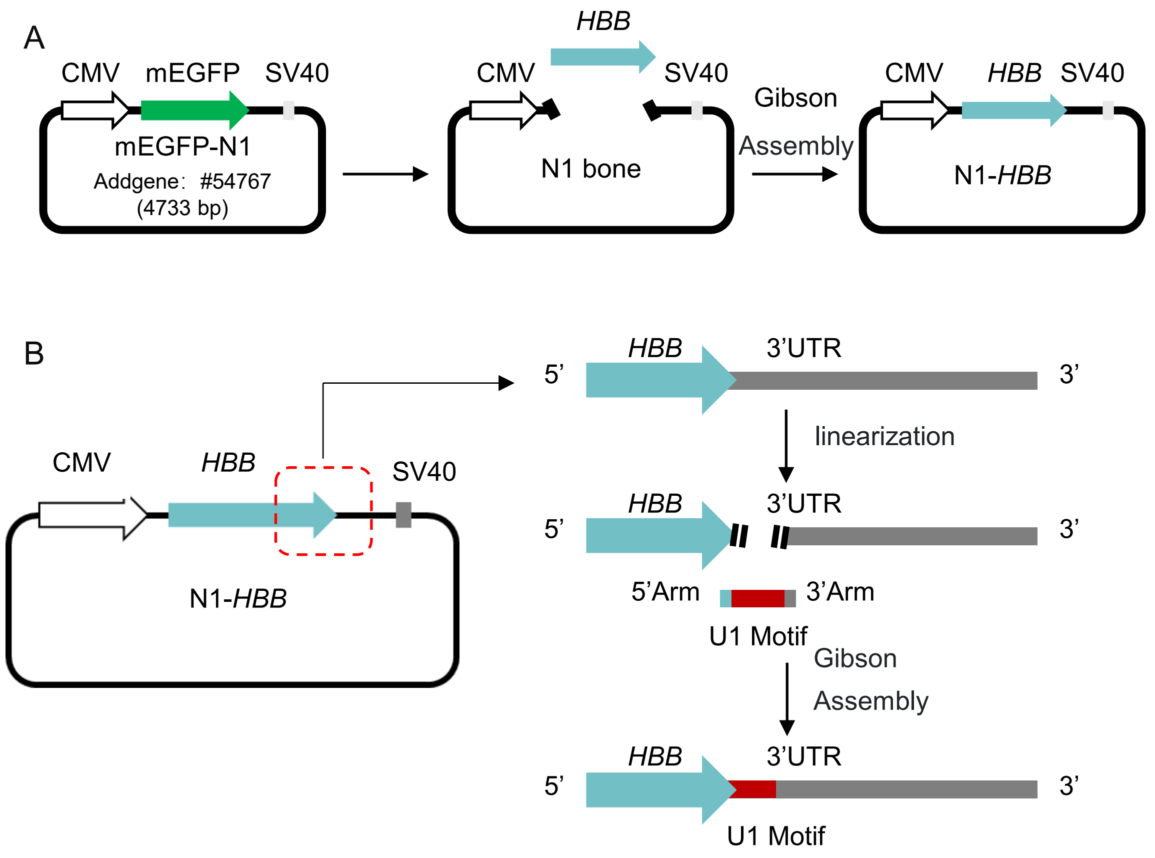

Figure S1. A. The procedure for constructing the *N1-HBB* plasmid from *mEGFP-N1 (Addgene: #54767)*; B. Schematic diagram illustrating the insertion of the U1 motif in the 3' UTR of the HBB gene in *N1-HBB*.

Figure S2.

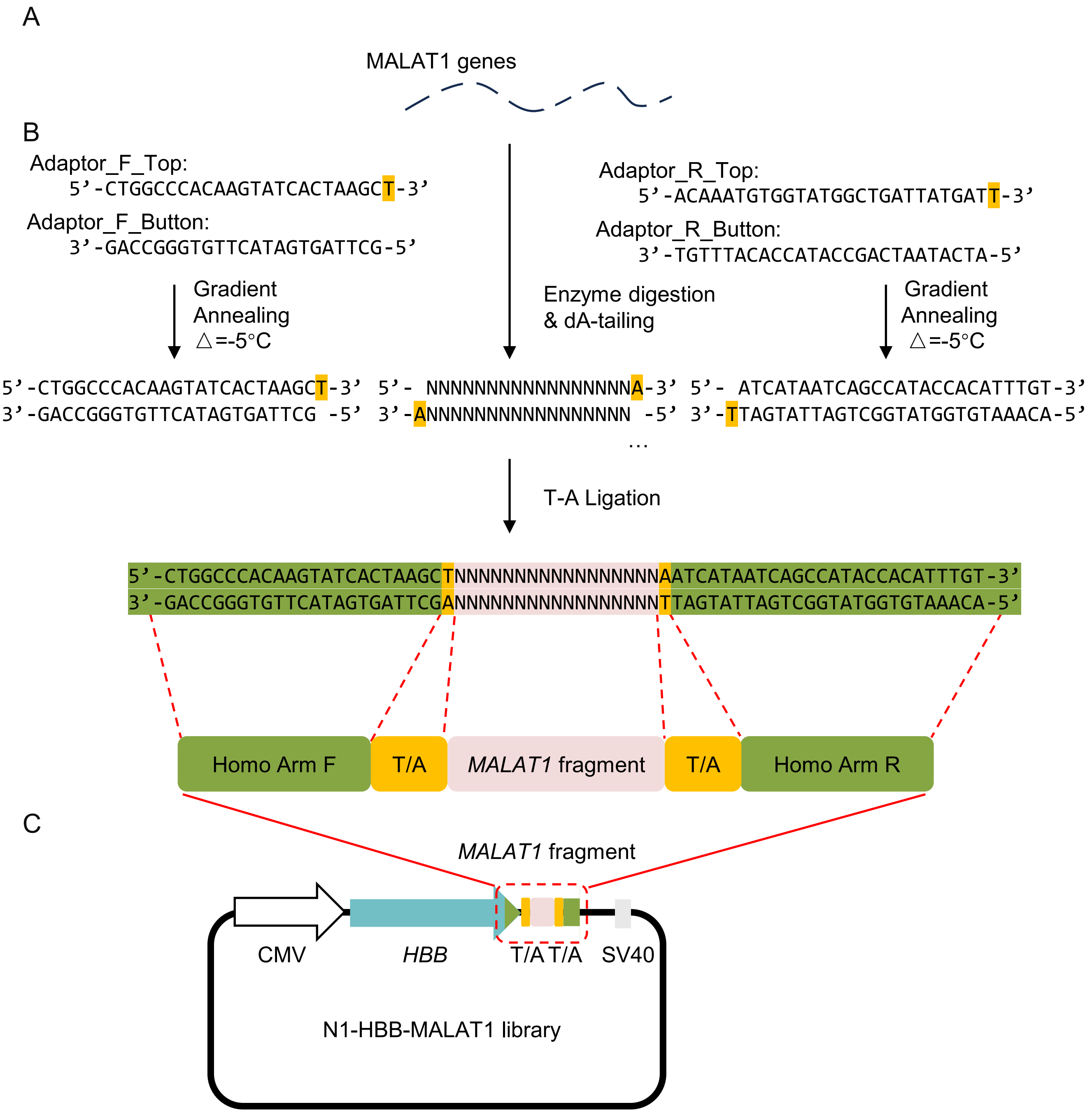

Figure S2. The procedure for constructing the *N1-HBB-MALAT1* plasmid library including random fragmentation (A), T-A ligation (B) and Gibson assembly (C).

Figure S3.

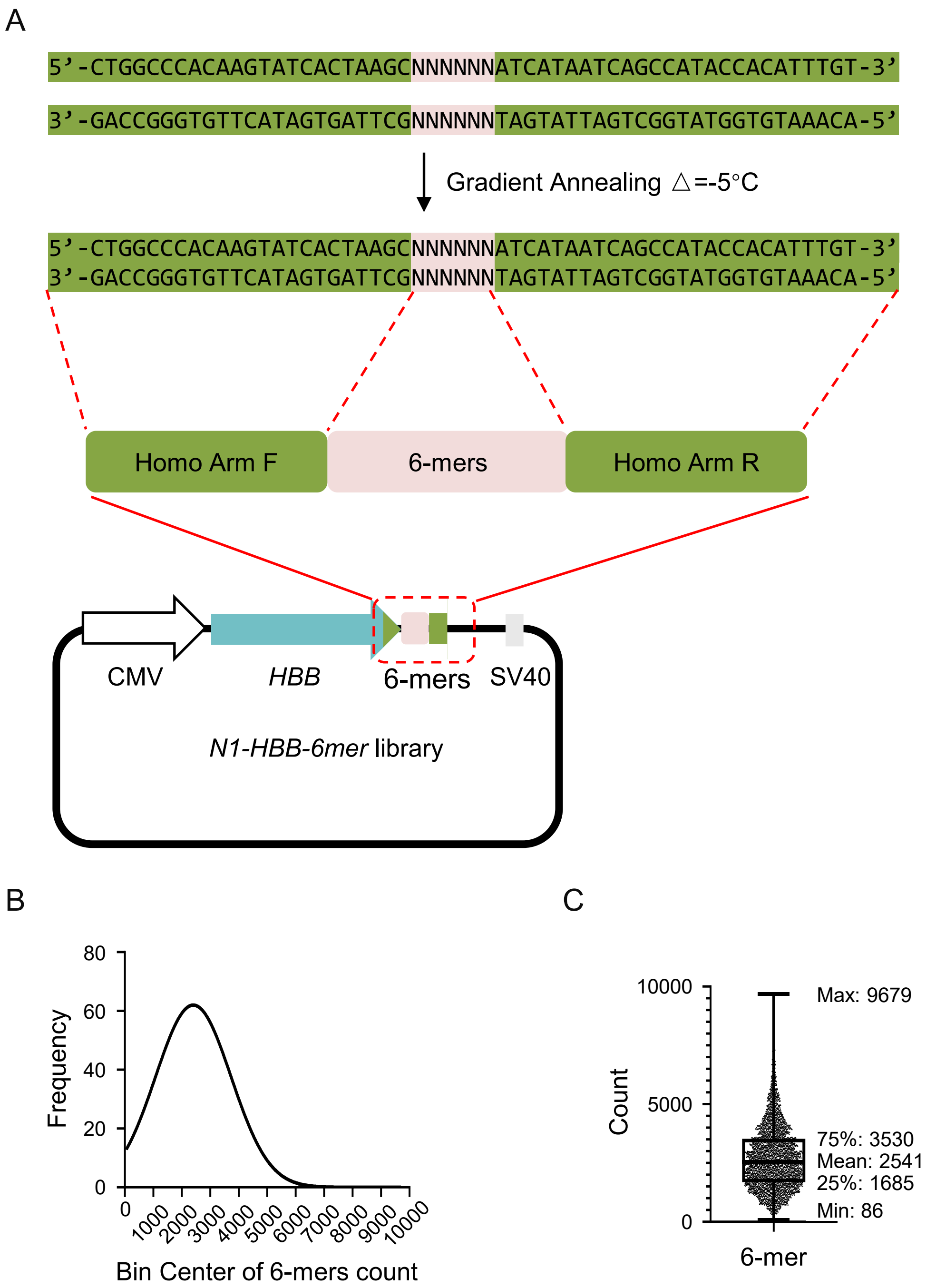

Figure S3. A. The procedure for constructing the *N1-HBB-6mer* plasmid library. B. The frequency of each 6-mer in the *N1-HBB-6mer* plasmid library. C. Violin plot showed the distribution of 6-mers in the *N1-HBB-6mer* plasmid library.

Figure S4.

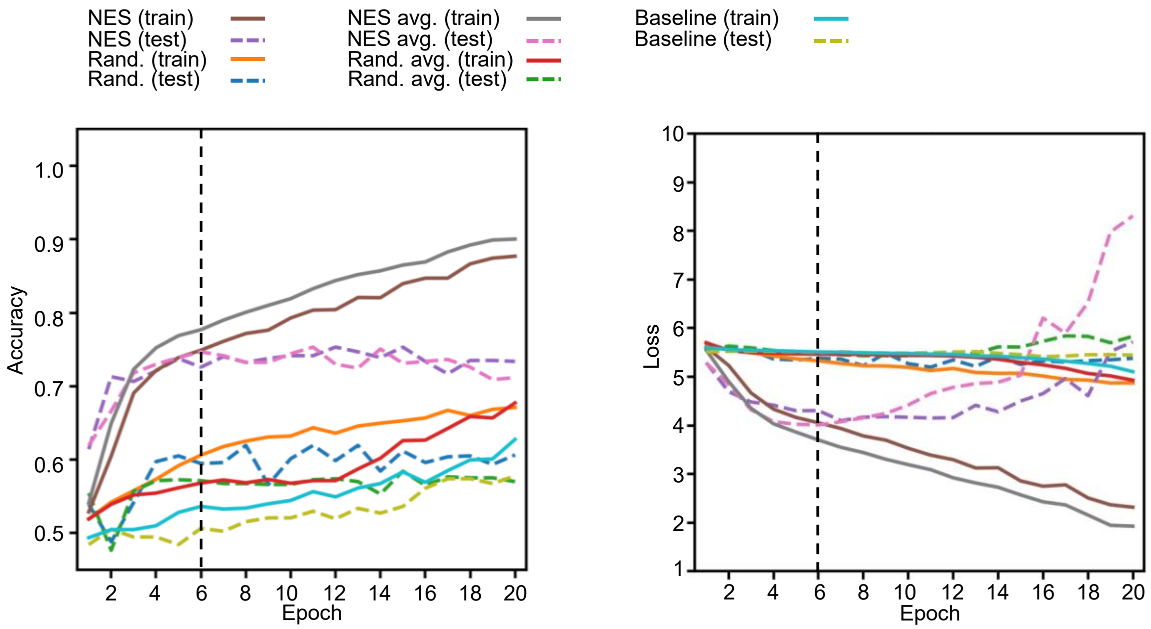

Figure S4. The overfitting evaluation of the NRS-guided deep learning model for RNA subcellular localization prediction. The epoch-accuracy curve in the training process (the right panel). The epoch-loss curve in the training process (the left panel). These analyses showed that the loss curve of the testing dataset using the model trained with NRS and NRS avg. turn to increase indicate overfitting, so we finished the training process at epoch 6.

Figure S5.

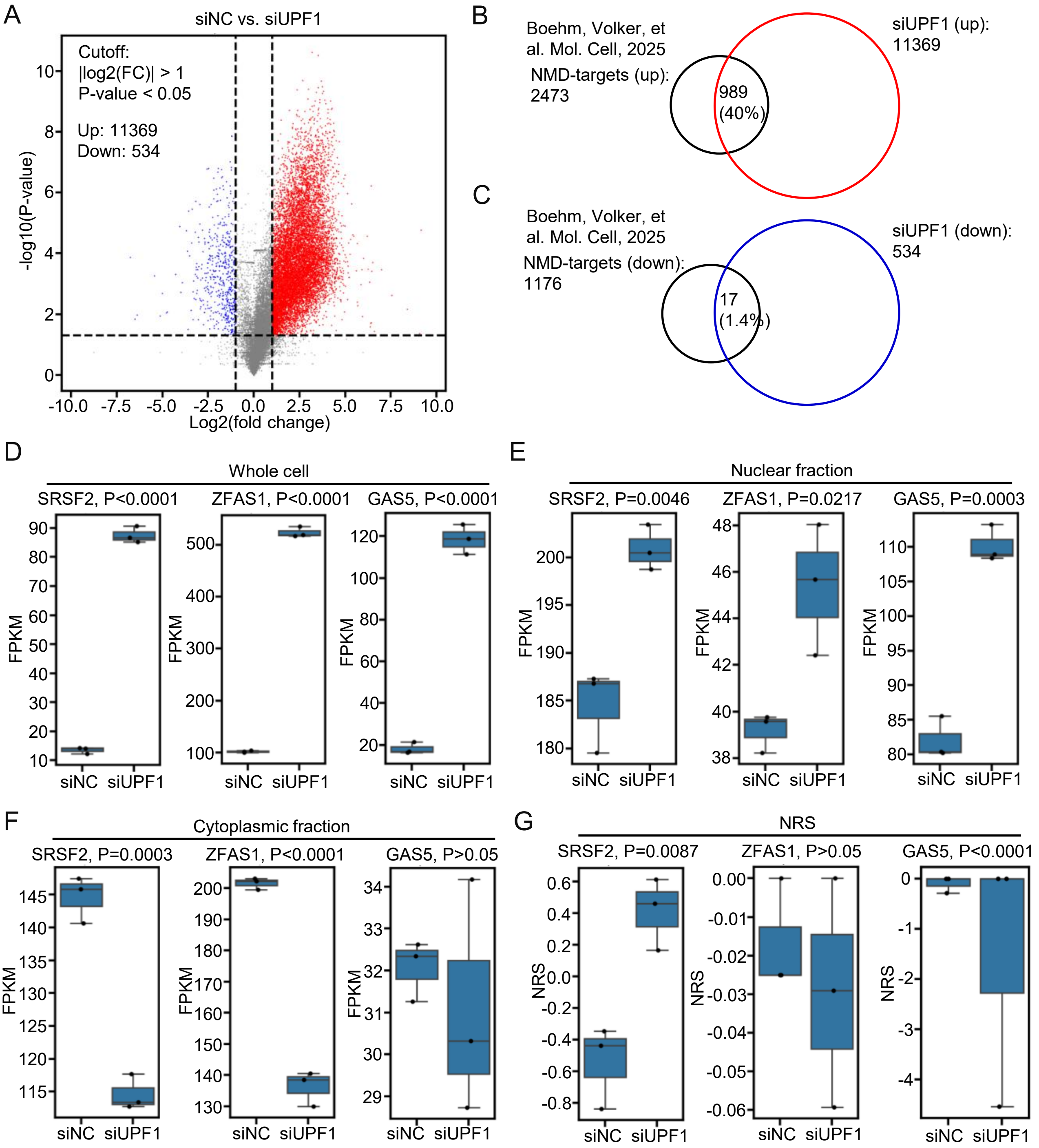

Figure S5. The expression of the Nonsense-mediated RNA decay (NMD)-targeted genes upon UPF1 knockdown. A. The volcano plot shows the up-regulated genes upon UPF1 knockdown. B and C. The up-regulated genes upon UPF1 knockdown are associated with the reported NMD-targeted genes (~40%). D-F. The expression of 3 canonical NMD-targets: SRSF2, ZFAS1 and GAS5 in whole cell, nuclear fraction and cytoplasmic fraction. G. The NRS of SRSF2, ZFAS1 and GAS5 upon UPF1 knockdown. We found that UPF1 knockdown does not drive cytoplasmic RNA accumulation.

Supplemental Tables

Table S1. List of PCR primers used in plasmid library construction.

Table S2. List of RT-qPCR primers used in this study.

Table S3. The datasets used in this study.

Table S4. The fragments associated subcellular localization in MALAT1.

Table S5. The list of nuclear- or cytoplasmic-enriched 6mers.

Table S6. The reported elements and the discoveried elements associated with RNA localization.

Table S7. The log_2(Nuc./Cyto.) profile of the nuclear- or cytoplasmic-enrich genes across 12 cell types.

Table S8. The log2(Nuc./Cyto.) of all transcripts upon knockdown of UPF1, YBX3 or DDX3X.
